# Case Report: A Rare Gastrojejunal Connection Causing Internal Hernia

**DOI:** 10.1002/ccr3.71718

**Published:** 2025-12-23

**Authors:** Jingjun Yang, Chenfei Jiang

**Affiliations:** ^1^ Department of General Surgery Haining People's Hospital Jiaxing Zhejiang China

**Keywords:** appendicitis, gastrojejunal connection, internal hernia, jejunum

## Abstract

Internal hernias are rare and often difficult to diagnose due to their nonspecific clinical presentation. We describe an unusual case of an internal hernia caused by an abnormal gastrojejunal connection, incidentally identified during a laparoscopic appendectomy. Intraoperative exploration revealed a fibrous band extending from the jejunal wall to the posterior gastric wall, creating a hernia orifice that entrapped the small intestine and its mesentery. The anomalous band was excised in its entirety, and the herniated bowel was repositioned to restore normal anatomy. The patient recovered uneventfully and remained asymptomatic during 1 year of follow‐up. This report highlights the diagnostic value of CT reconstruction and the importance of meticulous intra‐abdominal exploration in managing atypical internal hernias.

## Key Clinical Message

This rare case of internal hernia caused by an abnormal gastrojejunal connection was incidentally discovered during laparoscopic appendectomy, underscoring the importance of thorough intraoperative exploration to prevent missed diagnoses.

**Abbreviation**
CT: computed tomography

## 1 Introduction

Internal hernias are defined as the protrusion of a viscus through a congenital or acquired peritoneal or mesenteric aperture while remaining within the peritoneal cavity [1]. Although rare, with an incidence of less than 1% in the general population, they account for up to 6% of small bowel obstructions and may lead to bowel strangulation with mortality rates exceeding 50% if not promptly managed [2, 3]. Etiologies include congenital anomalies such as malrotation or abnormal peritoneal attachments and acquired causes such as surgical defects, trauma, or intra‐abdominal inflammation [4]. We report a rare case of an internal hernia caused by an abnormal gastrojejunal connection. This case underscores the critical importance of thorough intra‐abdominal exploration during laparoscopic procedures.

## 2 Case History/Examination

An 18‐year‐old female was admitted with a chief complaint of periumbilical pain that migrated to the right lower quadrant over the course of 1 day. The pain initially presented as intermittent, vague pain in the upper abdomen without any identifiable precipitating factors and was not associated with other gastrointestinal symptoms. Due to the mild nature of the discomfort, the patient did not initially seek medical attention. However, the pain subsequently migrated to the right lower quadrant and was accompanied by nausea and vomiting. The vomitus consisted of partially digested gastric contents, with no evidence of hematemesis. Abdominal pain was slightly relieved after vomiting but recurred and progressively intensified. The patient denied experiencing chills, fever, diarrhea, chest tightness, palpitations, urinary frequency, or urgency. The patient presented to our outpatient clinic for further evaluation and management. Abdominal ultrasonography of the right lower quadrant revealed a non‐compressible, tubular structure with hypoechoic walls, suggestive of acute appendicitis. Based on these results, a clinical diagnosis of acute appendicitis was established, and the patient was subsequently admitted for inpatient treatment. She denied any history of abdominal trauma, prior surgical interventions, or intra‐abdominal infections. She also reported no history of smoking or alcohol consumption. She is unmarried and nulliparous, with a history of sexual activity and consistent use of appropriate contraceptive methods. Her menstrual cycles were regular, with normal flow and no dysmenorrhea. She denied any family history of hereditary diseases. Both parents were in good health, and her one younger brother and three younger sisters were also healthy.

### 2.1 Physical Examination

Temperature: 36.5°C; Heart rate: 78 beats per minute; Respiratory rate: 18 breaths per minute; Blood pressure: 118/75 mmHg; Height: 158 cm; Weight: 43 kg. The patient was conscious and in good spirits. Pupils on both sides were sensitive to light. No jaundice of the skin or sclera was observed. The neck was supple, and no enlarged superficial lymph nodes were palpable. Cardiac and pulmonary examinations revealed no abnormalities. The abdomen was flat, with bowel sounds at three per minute. The abdominal wall was soft, and the liver and spleen were not palpable. Localized tenderness was noted at McBurney's point in the right lower quadrant, without rebound tenderness. The psoas sign was negative, and the colon inflation test was negative. No abnormal masses were palpable. Shifting dullness was negative. No costovertebral angle tenderness was noted. No edema of the lower extremities was observed, and pathological reflexes were negative.

## 3 Differential Diagnosis, Investigations and Treatment

Before imaging studies were performed, the patient's presentation of periumbilical pain migrating to the right lower quadrant, accompanied by nausea and vomiting, prompted consideration of several differential diagnoses. Acute appendicitis was the primary clinical suspicion given the characteristic migratory pain pattern. Peptic ulcer perforation was also considered, as it may initially present with upper abdominal pain accompanied by nausea and vomiting; after perforation, the pain may also radiate or migrate to the right lower quadrant. However, the absence of sudden severe onset or overt peritoneal signs made this diagnosis less likely at the time of evaluation. Early gastroenteritis remained a consideration, though the lack of diarrhea or systemic manifestations reduced its likelihood. Mesenteric lymphadenitis—often mimicking appendicitis in young patients—was also included in the differential. Because the patient was of reproductive age, gynecologic conditions such as ovarian torsion, ruptured ovarian cyst, and pelvic inflammatory disease were considered; however, the absence of pelvic pain, vaginal symptoms, or menstrual abnormalities made these conditions less probable. Urinary tract disorders, including ureteral calculi and urinary tract infection, were also evaluated, but were less likely given the lack of urinary symptoms and negative costovertebral angle tenderness. Although small bowel obstruction may present with abdominal pain and vomiting, the early clinical course and absence of abdominal distension rendered this diagnosis less likely during initial assessment.

Further evaluation revealed the following abnormalities: Complete blood count demonstrated leukocytosis, with a white blood cell (WBC) count of 10.8 × 10^9^/L (reference range: 3.5–9.5 × 10^9^/L), neutrophil count of 8.28 × 10^9^/L (1.8–6.3 × 10^9^/L), and neutrophil percentage of 76.6% (40%–75%). Abdominal ultrasonography of the right lower quadrant revealed a non‐compressible, tubular structure with hypoechoic walls, suggestive of acute appendicitis. Abdominal non‐contrast computed tomography (CT) revealed an appendicolith, mild thickening of the appendix, and surrounding fat stranding. These imaging features were consistent with acute appendicitis and were accompanied by a small volume of pelvic effusion.

**FIGURE 1:**
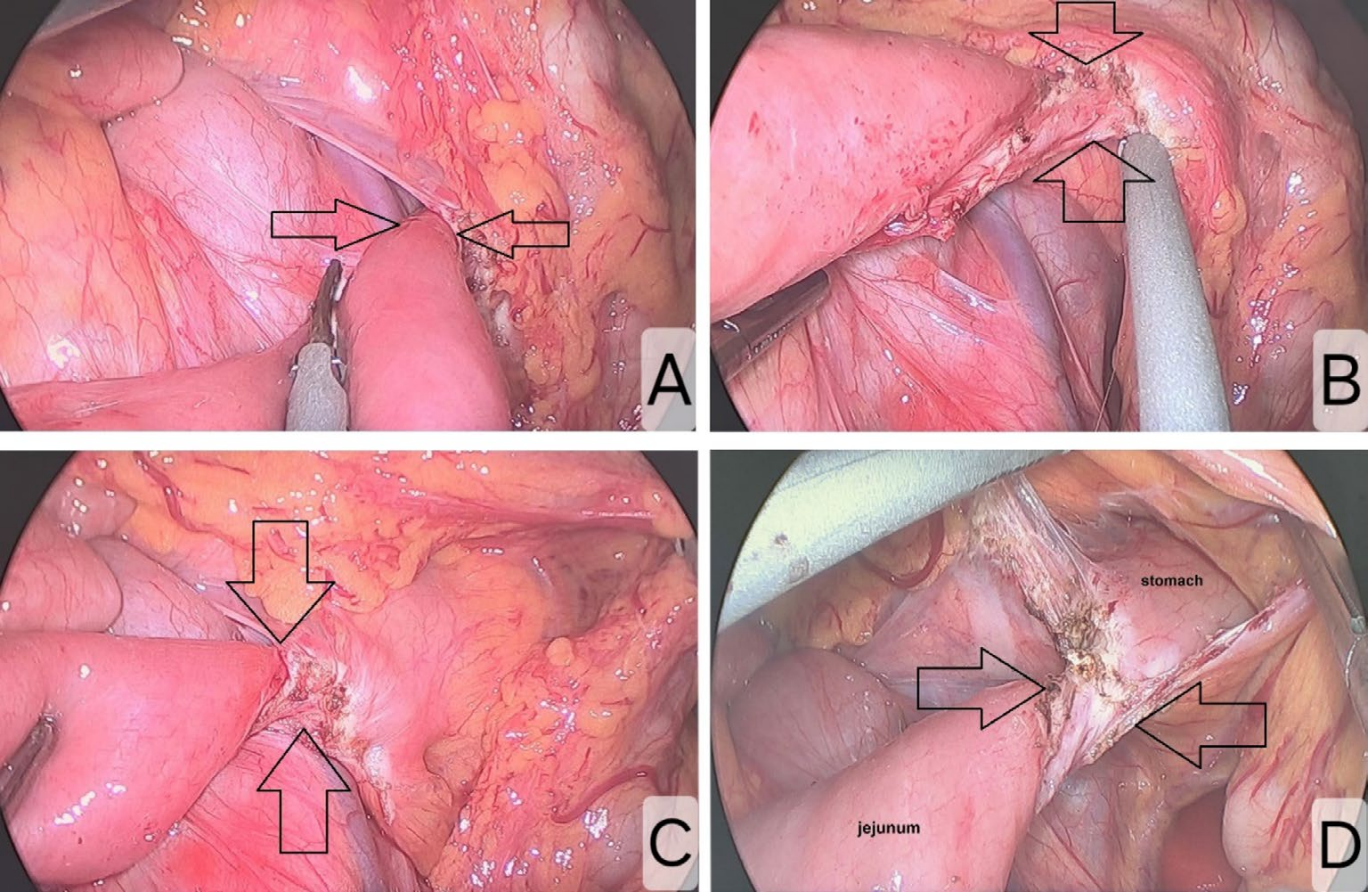
Gastrojejunal connection observed during surgery (arrow), anterior to the gastrojejunal connection (A), posterior to the gastrojejunal connection (B), elevation of the transverse colon (C), and incision of the transverse mesocolon (D).

A preoperative diagnosis of acute appendicitis was established. The patient subsequently underwent emergency laparoscopic appendectomy. During intraoperative exploration, an internal hernia was unexpectedly identified. After repositioning all small intestinal loops to the right side of the abdominal cavity, an abnormal jejunal structure was visualized. A fibrous band‐like connection was noted extending from the jejunal wall to the mesentery of the transverse colon. Upon careful dissection along this band, it was found to continue posteriorly toward the gastric wall, where it was fused with the posterior wall of the stomach (Figure 1). The connecting band was excised proximally near its attachment to the gastric wall and distally near the jejunal wall, resulting in complete resection. The hernial orifice was opened, and the incarcerated segment of small intestine along with its mesentery was released. The anatomical position of the small intestine and its mesentery was restored.

## 4 Outcome and Follow‐Up

The patient had an uneventful postoperative recovery and was discharged in stable condition without any complications.

**FIGURE 2:**
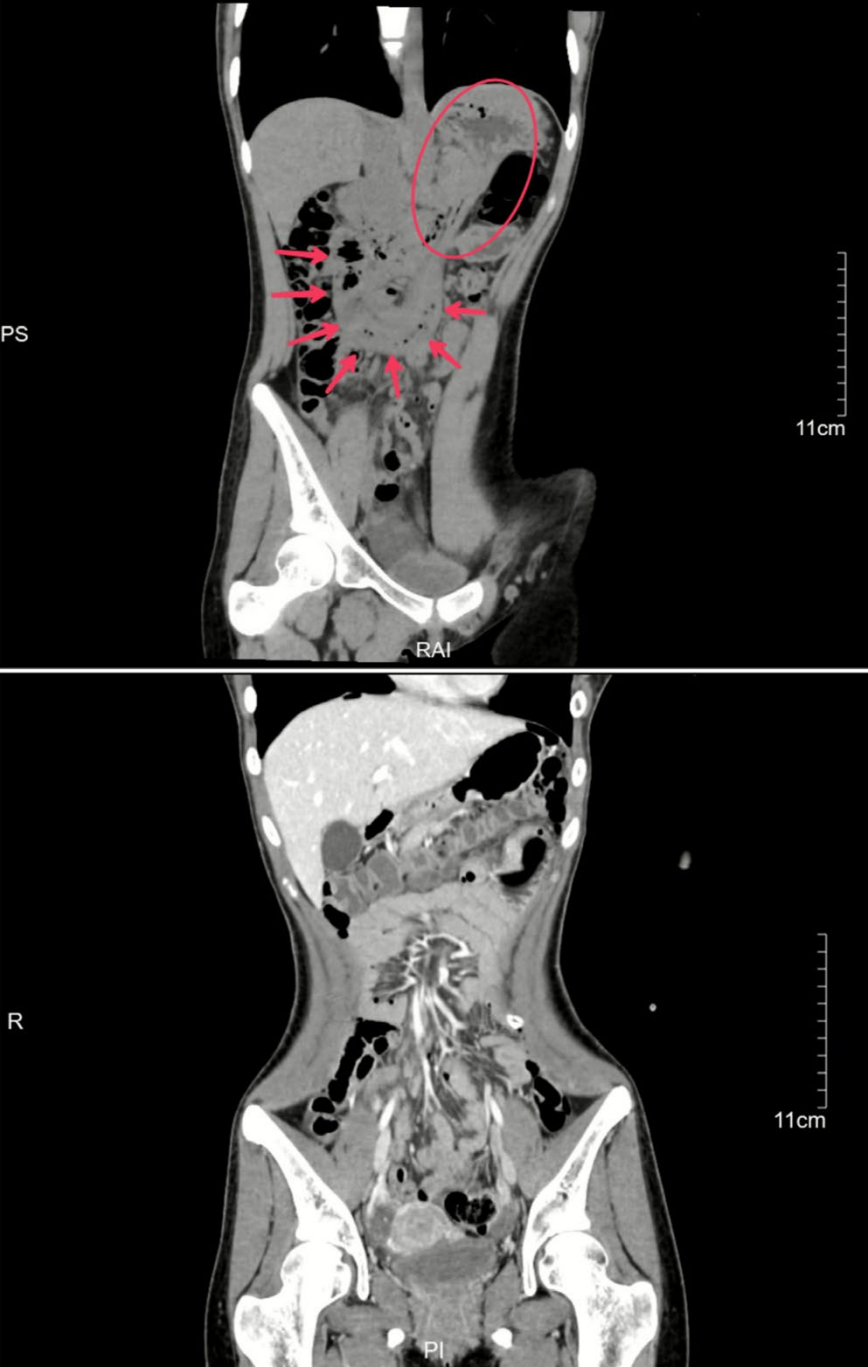
Comparison of coronal CT images before and after surgery: whirlpool‐like configuration of the small bowel (arrow) and associated traction on the gastric wall (circle).

Review of the preoperative abdominal CT scan, along with subsequent multiplanar reconstruction, revealed a gastrojejunostomy‐like structure in the coronal plane. A whirlpool‐like configuration of the small bowel and associated traction on the gastric wall were clearly visualized (Figure 2). At the time of this writing, the patient had been followed up by telephone for 1 year postoperatively, during which she reported no abdominal discomfort or other related symptoms.

## 5 Discussion

Hernias are broadly classified into two main types: external and internal [5]. External hernias involve the protrusion of intestinal loops through a defect in the abdominal or pelvic wall [6]. In contrast, internal hernias are defined as the herniation of a viscus through a normal or abnormal peritoneal or mesenteric aperture, while remaining confined within the peritoneal cavity [7]. The hernial orifice may be either acquired—resulting from previous surgery, trauma, or intra‐abdominal inflammation—or congenital, which includes both natural openings such as the foramen of Winslow and abnormal apertures due to anomalies of intestinal rotation and peritoneal attachment [8].

Internal hernias are further categorized based on anatomical location, following the classification proposed by Meyers [9]. The most common types include paraduodenal (53%), pericecal (13%), foramen of Winslow (8%), transmesenteric and transmesocolic (8%), intersigmoid (6%), and retroanastomotic (5%) hernias. The remaining 7% described by Meyers, including paravesical hernias, are not considered true internal hernias. The overall incidence of internal hernias ranges from 0.2% to 0.9%.

However, if intestinal obstruction or strangulation occurs, the consequences can be severe and potentially life‐threatening.

In the present case, the hernial orifice was created by an abnormal gastrojejunal connection. A significant portion of the small intestine and its mesentery became entrapped within this defect. This anatomical configuration constituted the pathological basis for potential bowel obstruction and strangulation. During surgery, the abnormal gastrojejunal connection was resected, the herniated contents were repositioned, and the hernia orifice was effectively eliminated, thereby preventing serious complications and achieving a favorable outcome.

The clinical presentation of internal hernias is highly variable, and symptoms are often atypical [8], particularly in emergency settings, which complicates timely diagnosis. In this case, no abnormalities were detected on axial CT imaging; however, postoperative coronal CT reconstruction revealed the presence of the hernia. This underscores the diagnostic value of multiplanar reconstruction techniques in suspected internal hernia cases. Moreover, previous studies have demonstrated that gastrointestinal contrast studies and contrast‐enhanced abdominal CT are effective tools for the early detection of internal hernias and should be considered in high‐risk patients [10].

This patient had no history of prior abdominal surgery, infection, or trauma, which argues against an acquired etiology. A congenital internal hernia is therefore more likely. Notably, the hernial configuration in this case did not correspond to any of the previously described types, suggesting a rare or possibly novel form of congenital internal hernia. Although internal hernias are relatively uncommon overall, the incidental identification of one during an appendectomy is exceedingly rare. In this case, the patient initially underwent laparoscopic appendectomy. Standardized abdominal exploration during the procedure led to the detection of the internal hernia, thereby avoiding a missed diagnosis. This case highlights the critical importance of thorough and systematic intra‐abdominal exploration during laparoscopic surgeries.

## 6 Conclusions

CT reconstruction is crucial for confirming internal hernias. Thorough intra‐abdominal exploration during laparoscopic surgery helps ensure accurate diagnosis and prevent missed intra‐abdominal anomalies in patients with atypical symptoms.

## Author Contributions

**Jingjun Yang:** writing – original draft, writing – review and editing. **Chenfei Jiang:** writing – review and editing.

## Funding

This work was supported by the Zhejiang Provincial Traditional Chinese Medicine Science and Technology Project (No. 2024ZL1100).

## Ethics Statement

The authors have nothing to report.

## Consent

Written informed consent was obtained from the patient's parents/guardians to publish this report in accordance with the journal's patient consent policy.

## Conflicts of Interest

The authors declare no conflicts of interest.

## Data Availability

Data available on request due to privacy/ethical restrictions.
